# Roles of *Salmonella typhi* and *Salmonella paratyphi* in Gallbladder Cancer Development

**DOI:** 10.31557/APJCP.2021.22.2.509

**Published:** 2021-02

**Authors:** Ratnakar Shukla, Pooja Shukla, Anu Behari, Dheeraj Khetan, Rajendra K Chaudhary, Yasuo Tsuchiya, Toshikazu Ikoma, Takao Asai, Kazutoshi Nakamura, Vinay K Kapoor

**Affiliations:** 1 *Department of Surgical Gastroenterology, Sanjay Gandhi Postgraduate Institute of Medical Sciences, Lucknow, India. *; 2 *Department of Transfusion Medicine, Sanjay Gandhi Postgraduate Institute of Medical Sciences, Lucknow, India. *; 3 *Division of Preventive Medicine, Niigata University Graduate School of Medical and Dental Sciences, Niigata, Japan. *; 4 *Department of Clinical Engineering and Medical Technology, Niigata University of Health and Welfare, Niigata, Japan. *

**Keywords:** Gall bladder cancer, gall stone, modified Widal test, Salmonella typhi, Salmonella paratyphi

## Abstract

**Background:**

Typhoid (*Salmonella typhi* and *paratyphi*) carriers and gall bladder cancer (GBC) are endemic in northern India. Results of previous studies about association of typhoid carriers with GBC are inconsistent. We studied antibodies against *Salmonella typhi* and *paratyphi* in serum samples of patients with GBC.

**Methods:**

We performed modified Widal test for antibodies against* Salmonella typhi* (Vi and O) and* Salmonella paratyphi* (AO and BO) antigens in patients with GBC (n=100), xanthogranulomatous cholecystitis (XGC, n=24), chronic cholecystitis (CC, n=200) and healthy controls (HC, n=200).

**Results:**

Serum antibodies against *Salmonella* were more frequently positive in GBC (22%) and XGC (29%), particularly in males in age ≥50 years (GBC: 47% and XGC: 50%) vs. HC (0) (P<0.01). Vi antibody was more common in GBC (13%, OR:9.8) and XGC (8%, OR:5.9) than HC (2%). O antibody was more common in GBC (8%, OR: 8.6) and XGC (8%, OR: 9.0) than HC (1%). O antibody was also more common in males with GBC (12%) than CC (1%) and HC (1%) (P=0.02 and P<0.001, respectively). AO (6%) and BO (4%) antibodies were detected in GBC, particularly in males, than HC (0), (P<0.01). *Salmonella* antibodies were more frequent in GBC with GS than those without GS (50% vs. 20%, OR=3.94, P=0.01).

**Conclusions:**

*Salmonella* carrier state was more common in GBC and XGC, particularly in elderly males than HC. The Vi antibody was more common in GBC and XGC than HC.* Salmonella* infection was more common in GBC with GS than those without GS.

## Introduction

Gall bladder cancer (GBC), commonest biliary tract cancer worldwide, has extremely poor prognosis because it is usually detected at an advanced stage due to delayed clinical presentation and metastasis to regional lymph nodes, liver and peritoneum (Kanthan et al., 2015). Incidence of GBC varies worldwide; GBC is not common in Western world like northern America (USA and Canada), Western Europe, Australia and New Zealand (Kapoor and McMichael, 2003). Higher frequency of GBC has been reported from Chile, Bolivia, Japan, Korea and China (Kapoor and McMichael, 2003). In India, North and North-East have higher incidence of GBC than South and West (Kapoor and McMichael, 2003). In urban Delhi, GBC is third leading cancer type in females with age adjusted rates (AAR) of 11.8/100,000 per year (Malhotra et al., 2017).

Prolonged exposure of gall bladder (GB) to gall stones (GS) and resultant chronic cholecystitis (CC) are most common risk factors for development of GBC (Dutta et al., 2019). Xanthogranulomatous cholecystitis (XGC) is a severe form of CC, which is considered to be a premalignant condition; XGC and GBC may coexist (Kapoor et al., 2016). Other common risk factors for GBC include ethnicity, genetic susceptibility, gender, age, lifestyle, GB polyps, bacterial infections and environmental factors (Tsuchiya et al., 2018; Dutta et al., 2019). 

Several studies have shown association of bacterial infections with increased risk of GBC (de Martel et al., 2009; Nath et al., 2010; Tsuchiya et al., 2018). Persistent bacterial infection leads to chronic inflammation via producing metabolites and toxins (Nath et al., 2010). These molecules may alter GB epithelium and lead to progression to GBC (Nath et al., 2010). Recent studies have shown association of *Salmonella typhi* and *Helicobacter pylori *with GBC (de Martel et al., 2009; Nagaraja and Eslick, 2014). *Salmonella enteric* serovar typhi (*Salmonella typhi*) and *Salmonella enterica* serotype paratyphi (*Salomonella paratyphi*) A are common etiological agents of typhoid and parathyphoid fever, respectively, while serotype *Salomonella paratyphi B* is rare (Pokhrel et al., 2009). *Salmonella typhi *infection is highly endemic in India. Annual incidence of *Salmonella typhi* is 110 per 1,00,000 population per year in India (Dutta et al., 2000). More interestingly, just as worldwide GBC incidence rate is one of the highest in India, *Salmonella typhi* infection rate is also quite high (Iyer et al., 2016).

It has been reported that chronic typhoid carriers carry higher risk of hepatobiliary cancers (Di Domenico et al., 2017). Subjects with biliary diseases like GS are at higher risk of *Salmonella typhi* infection (Dutta et al., 2000). Therefore, we hypothesized that typhoid carrier state is more likely to be related with GBC in subjects with GS than those without GS. Numerous studies have investigated relationship between *Salmonella typhi* and GBC but results are inconsistent (Dutta et al., 2000; Nath et al., 2008; Nagaraja and Eslick, 2014). This might be due to variation in the samples (serum, gall bladder tissue and bile) used for diagnostic procedures like culture, serological methods [enzyme linked immunosorbent assay (ELISA) and Widal test] and molecular methods [polymerase chain reaction (PCR) and real-time PCR]. Therefore, further investigation is needed to ascertain association of such infection with GBC. Culture methods of detecting *Salmonella typhi* in stool and bile samples are not only arduous but also less sensitive as chronic typhoid carriers often shed bacteria sporadically or excrete only small number of bacteria in stool (Ismail, 2000) and bile samples (Dutta et al., 2000). So, the objective of this study was to determine association of *Salmonella typhi*, *Salmonella paratyphi A* and *Salmonella paratyphi B *with GBC in serum samples using modified Widal test. 

## Materials and Methods

*Study participants and sample collection*

This is a collaborative study between Sanjay Gandhi Post Graduate Institute of Medical Sciences (SGPGIMS), Lucknow, India and Niigata University Graduate School of Medical and Dental Sciences (NUGSMDS), Niigata, Japan. We studied patients with GBC, XGC and CC, who attended Surgical Gastroenterology outpatient clinic of SGPGIMS from April’2017 to April’2019. We recruited only those subjects with GBC, XGC and CC who were positive by histopathology. Healthy controls (HC) were also included in the study to compare frequency of *Salmonella* infections between three study groups i.e. GBC, XGC and CC versus HC. Healthy controls were young, mostly male, voluntary blood donors. Two to three ml of whole blood was collected from each subject. Serum sample was separated and stored at -80^o^C for testing. Written informed consent was obtained from all study participants. Study protocol was approved by Institutional Ethics Committee (IEC) of both participating institutions (IEC codes for SGPGIMS, India: 2017-255-CP-101 and Niigata University of Health and Welfare, Japan: 17809-170517). 

*Modified Widal test*

Presence of serum antibodies against *Salmonella* antigens (Vi, O, AO and BO) was analyzed using commercially available *Salmonella* antisera kit (Denka Seiken Co., Ltd., Tokyo, Japan) in U-shaped 96 well microtiter plate (Schrader et al., 2008). Following agglutinin titers were considered as significant infection: *Salmonella* typhi Vi antigen: ≥20-fold dilution; *Salmonella* *typhi *O antigen: ≥160-fold dilution; O antigen for *Salmonella paratyphi A*: ≥80-fold dilution; and O antigen for *Salmonella paratyphi B*: ≥160-fold dilution (Schrader et al., 2008). All visible agglutination results were seen under slit lamp and scored from negative to 3+ (1+:only few cells clumped with cloudy background, 2+: 50% of cells clumped with moderately cloudy background, and 3+: 100% of cells clumped with clear background) (Schrader et al., 2008). 

*Statistical analysis*

All statistical analyses were performed using SPSS version 15.0 (SPSS Inc., Chicago, IL USA) and Graphpad Prism version 8.0 (GraphPad Software Inc, La Jolla, CA USA). Continuous data are represented as mean and standard deviation (SD). Chi-square and student’s t-test were used for categorical and continuous data, respectively. ANOVA with post hoc Bonferrani correction was applied for comparing more than two groups. Adjusted odds ratio (AOR) was calculated using binary logistic regression. Two tailed P-values less than 0.05 were considered as significant for all the statistical analyses. 

## Results

*Participant characteristics*

A total of 100 GBC, 24 XGC, 200 CC, and 200 HC were included. Mean age of patients with GBC was higher than that of CC and HC [GBC vs. CC (mean age, SD) = 52.3 (12.3) vs. 44.9 (13.4), P<0.001 and GBC vs. HC = 52.3 (12.3) vs. 32.2 (9.1), P<0.001), respectively]. However, mean age of patients with GBC and XGC was comparable [mean age (SD): 52.3 (12.3) vs. 54.4 (11.1), P=0.44]. In addition, mean age of female patients with GBC was higher than females with CC and HC [GBC vs. CC (mean age, SD) = 51.3 (12.0) vs. 44.1 (13.3), P<0.001 and GBC vs. HC = 51.3 (12.0) vs. 37.6 (6.7), P<0.05, respectively]. Mean age of male patients with GBC was higher than males with CC and HC [GBC vs. CC (mean age, SD): 55.2 (12.8) vs. 46.4 (13.6), P<0.01 and GBC vs. HC 55.2 (12.0) vs. 31.9 (9.2), P<0.001, respectively]. Gall stones were present in 20/69 GBC (information not available in remaining 31 patients), 24/24 XGC and 200/200 CC patients. 

*Prevalence of Salmonella infections in cases and controls*

Positive infection rates of *Salmonella* were higher in patients with GBC and XGC, particularly in males, than HC (P< 0.001) (Table 1). Odds ratio (OR) of males with GBC and XGC as compared to HC was 23.3 and 23.3, respectively (Table 1). Frequency of *Salmonella* infections in female patients with GBC and XGC were found to be comparable to female HC (P=0.19 and P=0.18, respectively) (Table 1). In addition, serum antibodies against *Salmonella* infections were more likely to be positive in patients with GBC and XGC in age group ≥50 years than HC (P=0.003 and 0.013, respectively) (Table 2). 

*Modified Widal test*

The frequency of Vi antibody detection was more in patients with GBC (13%, OR=9.8) and XGC (8%, OR=5.9) as compared to HC (2%) (P<0.001) (Table 3). Likewise, O antibody was more frequent in patients with GBC (8%, OR=8.6) and XGC (8%, OR=9.0) than HC (1%) (P=0.001 and P=0.01, respectively). O antibody was also more common in male patients with GBC than CC and HC (12% vs. 1%, P=0.02 and 12% vs. 1%, P<0.001, respectively). The AO (6%) and BO (4%) antibodies were more frequently detected in patients with GBC than HC (P=0.001 and P=0.004, respectively) but none of the HC were positive for these antibodies (Table 3). Moreover, AO and BO antibodies were more common in male patients with GBC and XGC than male HC (P<0.001) (Table 3). 

*Prevalence of Salmonella infections in patients with GBC with and without gall stones*

Out of 100 patients with GBC, 20 and 49 were with and without GS, respectively. We do not have the data regarding GS for the remaining 31 GBC subjects as they were diagnosed by cytopathology and were not operated. Rates of *Salmonella* infections were higher in GBC patients with GS than those without GS (50% vs. 20%, OR=3.94, 95% CI: 1.2-11.9, P=0.01). Moreover, frequency of *Salmonella* infections was more common in female GBC patients with GS (38%) than those without GS (14%); however, this difference was not statistically significant (OR=3.9, 95% CI=0.9-16.7, P=0.06). 

**Table 1:** Gender-Wise Distribution of Cases Positive for *Salmonella* Infections

	GBC		XGC		CC		HC		OR (95% CI) among different groups		AOR (95% CI)	P-value
	No.	Positive No. (%)	No.	Positive No. (%)	No.	Positive No. (%)	No.	Positive No. (%)				
Female	74	12 (16)	11	2 (18)	125	30 (24)	9	0	GBC vs. HC GBC vs. CC GBC vs. XGC XGC vs. HC XGC vs. CC	3.8 (0.2-69.7) 0.6 (0.3-1.3) 0.9 (0.2-4.5) 5.0 (0.2-118.7) 0.7 (0.1-3.4)		0.24
Male	26	10 (38)	13	5 (38)	75	21 (28)	191	5 (3)	GBC vs. HC GBC vs. CC GBC vs. XGC XGC vs. HC XGC vs. CC	23.3 (7.1-76.4) 1.6 (0.6-4.1) 1.0 (0.3-4.0) 23.3 (5.6-96.9) 1.6 (0.5-5.5)		< 0.001*
Total	100	22 (22)	24	7 (29)	200	51 (26)	200	5 (3)	GBC vs. HC GBC vs. CC GBC vs. XGC XGC vs. HC XGC vs. CC	11.0 (4.0-30.1) 0.8 (0.5-1.5) 0.7 (0.3-1.9) 16.1 (4.6-56.1) 1.2 (0.5-3.1)	0.0 (0.0-0.0) 0.6 (0.3-1.0) 1.0 (0.9-1.0) 0.0 (0.0-0.1) 1.0 (0.9-1.0)	< 0.001*

CC, Chronic cholecystitis; GBC, Gall bladder cancer; HC, Healthy controls; XGC, Xanthogranulomatous cholecystitis

**Table 2:** Positive Infection Rates of* Salmonella* in Cases and Controls in the Age Groups of <50 Years and ≥50 Years

	GBC				XGC		CC			HC			Comparisons among different groups	*P-value among subgroups	Overall *P-value
	Total No.	Positive No./Total No. (%)		Total No.	Positive No./Total No. (%)		Total No.	Positive No./Total No. (%)		Total No.	Positive No./Total No. (%)				
		<50 Years	≥50 Years		<50 Years	≥50 Years		<50 Years	≥50 Years		<50 Years	≥50 Years			
Female	74	5/31 (16)	7/43 (16)	11	2/6 (33)	0/5	125	17/79 (22)	13/46 (28)	9	0/8	0/1	GBC vs. HC GBC vs. CC GBC vs. XGC XGC vs. HC XGC vs. CC	NA 0.37 0.13 NA 0.23	0.28
Male	26	1/7 (14)	9/19 (47)	13	0/3	5/10 (50)	75	15/47 (32)	6/28 (21)	191	5/186 (3)	0/5	GBC vs. HC GBC vs. CC GBC vs. XGC XGC vs. HC XGC vs. CC	0.001 0.001 0.46 0.002 0.004	<0.001
Total	100	6/38 (16)	16/62 (26)	24	2/9 (22)	5/15 (33)	200	32/126 (25)	19/74 (26)	200	5/194 (3)	0/6	GBC vs. HC GBC vs. CC GBC vs. XGC XGC vs. HC XGC vs. CC	0.003 0.005 0.95 0.013 0.08	0.003

CC, Chronic cholecystitis; GBC, Gall bladder cancer; HC, Healthy controls; XGC, Xanthogranulomatous cholecystitis; *Chi-square test was used; NA, Not applicable as there is no positive case in HC

**Table 3:** Frequency of Serum Antibodies against *Salmonella Typhi* (Vi and O) and *Salmonella Paratyphi* (AO and BO) in Cases and Controls

	GBC		XGC				CC				HC				Comparisons among different groups		OR		95% CI		*P-value among different subgroups		Overall *P-value	
										
	No.	Positive No. (%)	No.		Positive No. (%)		No.		Positive No. (%)		No.		Positive No. (%)											
*Salmonella typhi *Vi antibody																								
Male	26	6 (23)^a^	13		0		75		14 (19)		191		3 (2)		GBC vs. HC GBC vs. CC GBC vs. XGC XGC vs. HC XGC vs. CC		18.8 1.3 8.6 1.9 0.16		4.4-81.0 0.4-3.8 0.4-165 0.0-40.7 0.0-2.8		< 0.001 0.63 0.06 0.65 0.09		< 0.001	
Female	74	7 (9)	11		2 (18)		124		16 (13)		9		0		GBC vs. HC GBC vs. CC GBC vs. XGC XGC vs. HC XGC vs. CC		2.1 0.7 0.5 5.0 1.5		0.1-40.1 0.3-1.8 0.1-2.6 0.2-118.7 0.3-7.6		0.33 0.48 0.38 0.18 0.61		0.54	
Total	100	13 (13)^a^	24		2 (8)^b^		200		30 (15)		200		3 (2)		GBC vs. HC GBC vs. CC GBC vs. XGC XGC vs. HC XGC vs. CC		9.8 0.8 1.6 5.9 0.5		2.7-35.3 0.4-1.7 0.3-7.8 0.9-37.7 0.1-2.3		< 0.001 0.64 0.53 0.03 0.38		< 0.001	
*Salmonella typhi *O antibody																								
Male	26	3 (12)^a,c^	13		1 (8)		75		1 (1)		191		2 (1)		GBC vs. HC GBC vs. CC GBC vs. XGC XGC vs. HC XGC vs. CC		12.3 9.7 1.6 7.9 6.2		1.9-77.7 0.9-97.4 0.1-16.7 0.7-93.1 0.4-105		0.001 0.02 0.71 0.054 0.15		0.004	
Female	74	5 (7)	11		1 (9)		125		6 (5)		9		0		GBC vs. HC GBC vs. CC GBC vs. XGC XGC vs. HC XGC vs. CC		1.5 1.4 0.7 2.7 1.9		0.1-29.4 0.4-4.9 0.1-6.9 0.1-75.1 0.2-18.2		0.42 0.56 0.78 0.35 0.54		0.75	
Total	100	8 (8)^a^	24		2 (8)b		200		7 (3)		200		2 (1)		GBC vs. HC GBC vs. CC GBC vs. XGC XGC vs. HC XGC vs. CC		8.6 2.3 0.9 9.0 2.5		1.7-41.3 0.8-6.8 0.2-4.8 1.2-67.1 0.4-12.8		0.001 0.09 0.96 0.01 0.25		0.012	
O antibody for *Salmonella* paratyphi A																								
Male	26	2 (8)^a^	13		2 (15) ^b^		75		10 (13)		191		0		GBC vs. HC GBC vs. CC GBC vs. XGC XGC vs. HC XGC vs. CC		39.1 0.5 0.5 83.3 1.2		1.8-838 0.1-2.7 0.1-3.7 3.8-1839 0.2-6.1		0.001 0.44 0.45 <0.001 0.84		< 0.001	
	GBC			XGC				CC				HC				Comparisons among different groups		OR		95% CI		*P-value among different subgroups		Overall *P-value	
										
	No.	Positive No. (%)		No.		Positive No. (%)		No.		Positive No. (%)		No.		Positive No. (%)											
O antibody for* Salmonella* paratyphi A																									
Female	74	4 (5)		11		0		125		10 (8)		9		0		GBC vs. HC GBC vs. CC GBC vs. XGC XGC vs. HC XGC vs. CC		1.2 0.7 1.5 NA 0.48		0.1-24.4 0.2-2.2 0.0-29.1 NA 0.0-8.7		0.47 0.49 0.43 NA 0.33		0.57	
Total	100	6 (6)^a^		24		2 (8)^b^		200		20 (10)		200		0		GBC vs. HC GBC vs. CC GBC vs. XGC XGC vs. HC XGC vs. CC		27.5 0.5 0.7 44.5 0.8		1.5-495 0.2-1.4 0.1-3.7 2.0-958 0.2-3.7		0.001 0.24 0.68 <0.001 0.79		0.001	
O antibody for *Salmonella* paratyphi B																									
Male	26	3 (8)^a^		13		2 (15) ^b^		75		5 (7)		191		0		GBC vs. HC GBC vs. CC GBC vs. XGC XGC vs. HC XGC vs. CC		57 1.8 0.7 83.2 2.5		2.9-1140 0.4-8.2 0.1-4.9 3.7-1839 0.4-14.8		<0.001 0.43 0.73 <0.001 0.28		0.001	
Female	74	1 (1)		11		1 (9)		125		6 (5)		9		0		GBC vs. HC GBC vs. CC GBC vs. XGC XGC vs. HC XGC vs. CC		0.4 0.3 0.1 2.7 1.9		0.0-10.2 0.0-2.3 0.0-2.4 0.0-75.1 0.2-18.2		0.73 0.2 0.11 0.35 0.54		0.42	
Total	100	4 (4)^a^		24		3 (12)^b^		200		11(5)		200		0		GBC vs. HC GBC vs. CC GBC vs. XGC XGC vs. HC XGC vs. CC		18.7 0.7 0.2 65.2 2.4		0.9-351 0.2-2.3 0.0-1.4 3.2-1307 0.6-9.5		0.004 0.57 0.1 <0.001 0.18		0.001	

CC, Chronic cholecystitis ; GBC, Gall bladder cancer; HC, Healthy controls ; XGC, Xanthogranulomatous cholecystitis;*Chi-square test was used; OR, Odds ratio; NA, Not applicable as there is no positive case in XGC vs. HC; ^a^P <0.01 between GBC vs. HC; ^b^P <0.05 between XGC vs. HC; ^c^P <0.05 between GBC vs. CC

## Discussion

The present study showed that prevalence of *Salmonella* infections was more frequent in patients ≥50 years of age with GBC and XGC, particularly in males, than HC. Among four (Vi, O, AO and BO) antibodies against *Salmonella* infections, Vi antibody was more frequent in GBC and XGC as compared to HC. Moreover, all four antibodies were found to be higher in patients with GBC and XGC than HC. More interestingly, frequency of *Salmonella* infections was more frequent in those GBC patients who had GS than those without GS.

Associations of *Salmonella* *typhi* and *Salmonella* *paratyphi* with GBC in present study are consistent with results from previous studies (Caygill et al., 1994a; Dutta et al., 2000; Sharma et al., 2007; Nath et al., 2008; Tewari et al., 2010; Nagaraja and Eslick, 2014). In India, frequency of serum antibodies against *Salmonella typhi *varies from 7.4% to 44.4% in patients with GBC based on ELISA, Widal or indirect hemagglutination assay (IHA) test (Dutta et al., 2000; Shukla et al., 2000; Vaishnavi et al., 2005; Sharma et al., 2007; Tewari et al., 2010; Nagaraja and Eslick, 2014), which is in accordance with results of our study (22%). Studies from Chile and Bolivia showed seropositivity rates of *Salmonella typhi* to be 15.4% and 46.7%, respectively (Strom et al., 1995b; Koshiol et al., 2016). Additionally, previous studies showed contribution of *Salmonella paratyphi *in pathogenesis of GBC (Ristori et al., 1982a; Caygill et al., 1994b; Singh et al., 1996). Nath et al. reported incidence of* Salmonella typhi *and *Salmonella paratyphi A* in bile samples among patients with GBC to be 10.7% and 3.5%, which is significantly higher (P=0.04) as compared to subjects with CC and HC (Nath et al., 1997). Frequency of typhoid carriers is more in patients with CC. The 26% prevalence of serum antibodies against *Salmonella* in patients with CC in our study is in accordance with findings of previous studies (Karaki, 1984; Vaishnavi et al., 2005). Chronic typhoid carrier state causes not only stone formation in GB but also leads to progression to carcinoma (Nagaraja and Eslick, 2014). Moreover, chronic typhoid carriers increase risk of GBC 8.47 fold than those who had acute or cleared infection (Shukla et al., 2000). Concomitantly, we found that association of *Salmonella* infection with GBC was 11 times higher than HC. This finding also agrees with results reported by Nath et al (Nath et al., 2008). Another study from Bolivia and Mexico reported that the incidence of GBC increases 12-fold in subjects with previous history of typhoid fever (Strom et al., 1995a). This indicates that early detection and clearance of typhoid carrier state is very important to prevent progression to GBC. 

Serum antibodies against *Salmonella* infection were more frequently positive in male patients with GBC (OR=23.3) and XGC (OR=23.3) as compared to HC. Nath et al. reported that males were 89 times more likely to be associated with typhoid carrier state (Nath et al., 2008). They showed that 90% of male patients in age group of 31-60 years were typhoid carriers (Nath et al., 2008). This may be explained by the fact that in India, males are more likely to go for outdoor activities and get exposed to environmental risks as compared to females who more often stay at home. We found that Vi antibodies were more likely to be present in patients with GBC (13%) and CC (15%), particularly in male patients, than HC (2%). A meta-analysis reported that prevalence of Vi antibody in patients with GBC and CC was 15.4% (6/39) and 5% (2/40), respectively (Koshiol et al., 2016). Another study had shown that frequency of Vi antibody was 7.4% and 10.2% in patients with GBC and GS, respectively (Vaishnavi et al., 2005). A study from Chile reported that frequency of *Salmonella paratyphi A* and *Salmonella paratyphi B* was 1.5% and 8.8% in a total of 1,000 bile samples (Ristori et al., 1982b). But studies regarding serological status of *Salmonella paratyphi A *and *B* in patients with GBC are lacking and, hence more studies need to be performed to draw any conclusions. 

There is a close association between typhoid carrier state and GS in patients with GBC. It is believed that the presence of GS along with typhoid carrier state represents a major risk factor for GBC (Caygill et al., 1994b; Di Domenico et al., 2017). This corresponds with results of present study in that typhoid carriers were detected more frequently in those GBC cases who had GS (50%) than those without GS (20%). *Salmonella typhi* is well known to form a biofilm on the gall stones (Di Domenico et al., 2017). *Salmonella typhi* is also supposed to reduce immune response in GB mucosa (Koshiol et al., 2016). Thus, a low inflammatory environment facilitates persistence of *Salmonella typhi* in the GB and formation of biofilms upon interaction with cholesterol or related substances (Koshiol et al., 2016). During chronic exposure, *Salmonella typhi* produces several carcinogenic substances including secondary bile acids, nitroso compounds and cytolethal distending toxin (CDT) (Di Domenico et al., 2017). Bacterial glucoronidase produces a number of intermediate compounds via acting on bile (Nath et al., 2008). These intermediate compounds and CDT act as a genotoxin with immunomodulatory effect and damage DNA of infected cells (Koshiol et al., 2016; Di Domenico et al., 2017). These compounds also act as tumor promoters and lead to carcinogenic alterations in GB mucosa that progress to GBC (Shukla et al., 2000; Koshiol et al., 2016; Di Domenico et al., 2017). Moreover, typhoid carriers lead to increased secretion of free radicals in GB tissues which has been shown to decrease after antibiotic administration (Nagaraja and Eslick, 2014). 

Association of *Salmonella typhi *with the carcinogenesis of GBC is widely known (Dutta et al., 2000; Tewari et al., 2010; Nagaraja and Eslick, 2014). But, there are some studies which have not reported an association between *Salmonella* infections and GBC. A study from Santiago, Chile reported no association between *Salmonella typhi* and GBC [OR: 1.12 (0.26–4.74)] (Csendes, 1994). Another study from Shanghai, China also did not find any association between *Salmonella typhi* and biliary tract cancer (Safaeian et al., 2011); prevalence of *Salmonella typhi* in biliary tract cancer, biliary-stone cases and population controls were 0.26%, 0.41% and 0.12%, respectively (Safaeian et al., 2011). These data indicate that there are other factors that are likely to contribute to development of GBC. Therefore, we need to further explore putative mechanism of GBC in relation to *Salmonella*. 

The key strength of our study is that this case-control study was performed in a large number of samples, particularly in a typhoid endemic region where incidence of GBC is also high. Larger sample size also enabled us to reduce result bias among different study subgroups. We have used modified Widal test, which is highly sensitive and specific for detection of *Salmonella antigens *(Schrader et al., 2008). Most importantly, we also showed the frequency of *Salmonella paratyphi A* and *B* antigens in serum samples of subjects with GBC and controls. Our study has some limitations also. Modified Widal test alone may not be efficient method for detection of *Salmonella* antigens. Therefore, higher sensitive and specific molecular methods like PCR or real-time PCR in association with modified Widal test would be ideal for serotyping of *Salmonella*e to avoid false positivity and negativity. 

In summary, we found that frequency of typhoid carriers and Vi antibody was higher in subjects with GBC and CC, particularly in males as compared to HC. Most importantly, we found that GBC cases with GS were more prone to *Salmonella* infections than those without GS. Thus, typhoid carrier state with GS increased the risk of GBC. Detection and eradication of typhoid carrier state in subjects with GS using either antibiotic administration or preventive cholecystectomy may help to prevent the progression to GBC, particularly in North India where prevalence of *Salmonella* infections is quite common. 
